# Dynamic peripheral blood immune cell markers for predicting the response of patients with metastatic cancer to immune checkpoint inhibitors

**DOI:** 10.1007/s00262-022-03221-5

**Published:** 2022-06-04

**Authors:** Chen Wei, Mengyu Wang, Quanli Gao, Shasha Yuan, Wenying Deng, Liangyu Bie, Yijie Ma, Chi Zhang, Shuyi Li, Suxia Luo, Ning Li

**Affiliations:** 1 Department of Internal Medicine, The Affiliated Cancer Hospital of Zhengzhou University & Henan Cancer Hospital, 127 Dongming Road, Zhengzhou, 450008 Henan China; 2 Department of Immunotherapy, The Affiliated Cancer Hospital of Zhengzhou University & Henan Cancer Hospital, 127 Dongming Road, Zhengzhou, 450008 Henan China

**Keywords:** Biomarkers, Oncology, Machine learning, Prognosis, Immunotherapy

## Abstract

**Purpose:**

Immune checkpoint inhibitors (ICIs) have shown durable responses in various malignancies. However, the response to ICI therapy is unpredictable, and investigation of predictive biomarkers needs to be improved.

**Experimental design:**

In total, 120 patients receiving ICI therapy and 40 patients receiving non-ICI therapy were enrolled. Peripheral blood immune cell markers (PBIMs), as liquid biopsy biomarkers, were analyzed by flow cytometry before ICI therapy, and before the first evaluation. In the ICI cohort, patients were randomly divided into training (*n* = 91) and validation (*n* = 29) cohorts. Machine learning algorithms were applied to construct the prognostic and predictive immune-related models.

**Results:**

Using the training cohort, a peripheral blood immune cell-based signature (BICS) based on four hub PBIMs was developed. In both the training and the validation cohorts, and the whole cohort, the BICS achieved a high accuracy for predicting overall survival (OS) benefit. The high-BICS group had significantly shorter progression-free survival and OS than the low-BICS group. The BICS demonstrated the predictive ability of patients to achieve durable clinical outcomes. By integrating these PBIMs, we further constructed and validated the support vector machine-recursive and feature elimination classifier model, which robustly predicts patients who will achieve optimal clinical benefit.

**Conclusions:**

Dynamic PBIM-based monitoring as a noninvasive, cost-effective, highly specific and sensitive biomarker has broad potential for prognostic and predictive utility in patients receiving ICI therapy.

**Supplementary Information:**

The online version contains supplementary material available at 10.1007/s00262-022-03221-5.

## Introduction

Cancer immunotherapies, mainly represented by immune checkpoint inhibitors (ICIs) that target the programmed death 1 ligand (PD-L1)/PD-1 axis, have shown promising efficacy in the treatment of many types of cancer [1]. However, only a subset of patients show durable responses, and reliable immune-related biomarkers that can effectively identify these patients before or early during treatment have remained elusive [2, 3]. Numerous studies have shown that patients with positive PD-L1 expression have a high response rate to ICIs [4, 5]. Moreover, a high tumor mutation burden (TMB) predicts ICI response across different types of cancer [6]. In addition to PD-L1 and TMB, various other biomarkers have been described for predicting response to ICIs, including IFN-*γ* signatures, proportion of CD8 + T cells, and genomic instability, as defined by microsatellite instability (MSI) [7–10]. Although tumor sampling is extensively employed for biomarker identification, obtaining tissue can be challenging owing to spatial heterogeneity, low tumor content, and difficulty with accessibility, resulting in lower than expected inter-observer concordance.

The evaluation of blood at baseline and on-treatment provides insights into the patient’s immune profile and how this relates to the ICI response [11]. Several studies have shown that peripheral blood immune cell markers (PBIMs), as liquid biopsy biomarkers, offer a promising approach for predicting ICIs therapy response [12, 13]. The frequencies of effector cells at baseline or during treatment tend to be correlated with better response, while high frequencies of suppressor cells such as regulatory T cells (Treg) are often associated with poor treatment outcomes [14]. However, the prognostic role of dynamic PBIMs and the comprehensive relationship between integrated PBIMs and the clinical response to ICIs have not been well established.

Here, we focused on PBIMs before and after therapy with PD-1 blocking antibodies in patients with metastatic cancer. We screened immunotherapy-related PBIMs associated with patient prognosis using the Cox regression model with a least absolute shrinkage and selection operator (Lasso) penalty to build a peripheral blood immune cells-based signature (BICS). Next, we successfully divided patients into two subgroups (high- and low-BICS subgroups). The high-BICS subgroup was not only found to be associated with poor prognosis, but also with no durable response to ICI therapy. Furthermore, we verified that BICS serves as an effective tool to identify patients with stable disease (SD) at the first scan who will eventually achieve durable clinical benefit (DCB). Finally, we established the SVM-RFE classifier model, which allows early identification of patients who are most likely to achieve optimal clinical benefit from ICI therapy.

## Materials and methods

### Patients

**Fig. 1:**
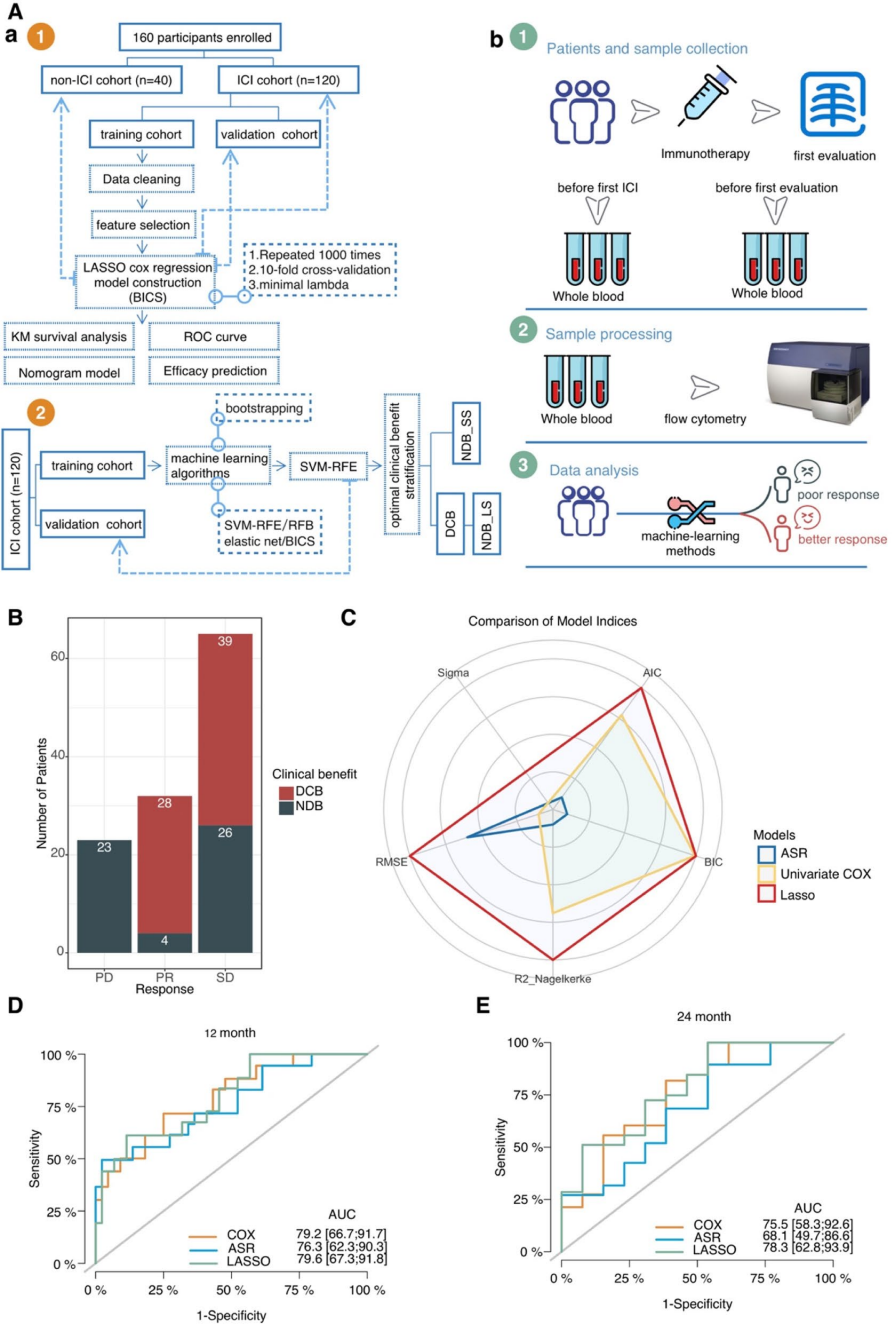
**A** Study overview. **a** Consort diagram of patients with metastatic cancer treated with ICI. **b** The pipeline for analysis of patient samples. **B** In the ICI cohort, of the 65 patients who achieved SD at the first scan, approximately 40% (*n* = 26) did not ultimately reach DCB. **C** The five common model indices, including AIC (Akaike’s Information Criterion), BIC (Bayesian Information Criterion), R2 (r-squared value), R2_adj (adjusted r-squared), and RMSE (root-mean-squared error) are normalized, and the Lasso model indicates better model performance than the ASR and Cox regression models. The weighted residuals of Cox Proportional Hazards (CoxPH) cannot be calculated, so we couldn't obtain the residual standard deviation (SIGMA) in CoxPH. (D) Time-dependent ROC curve at 1-year OS for the ASR, Lasso, univariate Cox regression in the training cohort (ASR AUC = 0.763, Lasso AUC = 0.796, and univariate Cox regression AUC = 0.792). (E) Time-dependent ROC curve at 2-year OS for the ASR, Lasso, and univariate Cox regression in the training cohort (ASR AUC = 0.681, Lasso AUC = 0.783, and univariate Cox regression AUC = 0.755). ICIs: immune checkpoint inhibitors; BICS: peripheral-blood-immune-cell-based signature; OS: overall survival; DCB: durable clinical outcomes; NDB: no durable benefit; NDB_LS: long-term survival with no durable clinical benefit; NDB_SS: short-term survival with no durable clinical benefit; PD: progressive disease; PR: partial response; SD: stable disease; LASSO: least absolute shrinkage and selection operator; ASR: all-subsets regression; AUC: area under the curve; ROC: receiver operating characteristic

The retrospective study design is given in Fig. 1A. The ICI cohort participants were patients with recurrent and/or metastatic cancer who were treated with ICIs in the Affiliated Tumor Hospital of Zhengzhou University between June 2018 and February 2021. The peripheral blood samples for immune phenotyping of patients treated with ICIs were collected at baseline and before the first scan. Additionally, the peripheral blood samples of patients receiving chemotherapy and/or targeted therapy without ICIs were collected in the non-ICI cohort at baseline and before the first scan for controlled validation. Patients could be enrolled independent of cancer entity. Patients were excluded if they did not have enrolled samples, or if they lacked information on tumor response assessment or survival follow-up.

### Assessment of clinical outcomes

The clinical treatment response after anti-tumor therapy was identified as SD, progressive disease (PD), or partial response (PR) according to the Response Evaluation Criteria in Solid Tumors (RECIST), version 1.1 [15]. Patients were categorized into DCB, defined as PR or SD with progression-free survival (PFS) ≥ 6 months, and no durable benefit (NDB). For the ICI cohort, PFS was defined as the time from the start of anti-PD-1 therapy to the first disease progression or death from any cause. Overall survival (OS) was evaluated as the time from the start of anti-PD-1 therapy until death from any cause. Patients with RECIST PD at the first scan subsequently continued with ICI treatment. For the non-ICI cohort, PFS and OS were defined as the time from the start of the systematic anti-tumor therapy. The list of detailed patient information is displayed in Table S4.

### Flow cytometry and data cleaning

Whole blood samples were collected from patients in the ICI cohort before ICI therapy and after two cycles of ICI. Peripheral blood was also collected from non-ICI patients before the treatment and before the first evaluation. Peripheral blood mononuclear cells (PBMCs) were isolated and PBIMs were determined by flow cytometry. Cell surface staining was performed for 30 min at 4 °C with the antibodies listed in Table S5. Data acquisition was conducted with a BD FACSCanto II, and data analysis was performed using FlowJo software.

We compared the differential variations (before the first evaluation minus before the treatment value; DV) of each PBIM. Considering the dynamics of changes in the immune status, we incorporated the DV of PBIMs along with PBIMs as variables in the subsequent analysis. Considering the strong correlation between the variables, we performed preliminary cleaning of the data. First, the “nearZeroVar” function in the Caret package was used to identify near zero-variance variables. Second, the “findCorrelation” function (Caret package) was applied to remove variables that were strongly correlated with other independent variables. Finally, the values of the remaining variables were quantile normalized and log2 transformed.

### Construction of the BICS

1. To assess the early forecasting benefit of PBIMs for binary classification (DCB vs NDB), the ICI cohort was randomly divided into training and validation cohorts, with the percentage of 0.75 to training using the Caret package.
2. We applied three well-established feature selection models (all-subsets regression (ASR); Lasso regression; and univariate Cox regression) to predict the prognosis. A detailed description of the Lasso and ASR regression is shown in the Supplementary Method section.
3. The performance of the three models was assessed based on two evaluation methods: (1) The five common model indices, including Akaike’s Information Criterion (AIC), Bayesian Information Criterion (BIC), r-squared value (R2), adjusted r-squared (R2_adj), and root-mean-squared error (RMSE), were computed using the “compare_performance” function in the performance package [16]; We used the function plot () for compare_performance () to creates a “spiderweb” plot, where the different indices are normalized and larger values indicate better model performance[16]; (2) the time-dependent area under the receiver operating characteristic (ROC) curve (AUC) of the three different models was calculated using the timeROC R package. Then, the Lasso regression was selected for the prognostic prediction model.
4. The predictive performance of three penalized regressions (Lasso regression, ridge regression, and elastic net) was assessed by Harrell’s concordance index (c-index). Finally, we obtained the best penalized regression model (Lasso) with the highest c-index, which was selected for the prognostic prediction model.

### Validation of the BICS

The survival curves were drawn using the Kaplan–Meier methods, and the log-rank test was used to evaluate statistical significance. On the basis of the correlation between BICS and the patients’ OS, the cut-off point of each database subgroup was determined using the survminer R package. The “surv-cutpoint” function, which repeatedly tests all potential cut points to establish the maximum rank statistic, was applied to dichotomize the BICS, and then patients were divided into high- and low-BICS groups. The time-dependent AUC was calculated using the timeROC R package. The nomogram plot was constructed using the rms R package.

### Construction and validation of the optimal clinical benefit predictor by multiple machine learning methods

The endpoints of optimal clinical benefit were used to achieve a binary classification, and the PBIMs were used as features for binary classification. The train-validation division was stratified by a ratio of 0.75. First, in the training cohort, elastic net, SVM-RFE, and random forest and Boruta (RFB) analyses with fivefold cross-validation were performed to determine the optimal features for each model via the Caret package in R. Elastic Net regression is a hybrid classification algorithm that blends both penalizations of the L2 and L1 regularization of lasso and ridge methods. SVM-RFE is a state-of-the-art algorithm that is used for gene selection, and it is a good choice to avoid overfitting when the number of features is high [17]. Random forest is an ensemble classification scheme that utilizes a majority vote to predict classes based on the partition of data from multiple decision trees. The Boruta algorithm was developed to identify all relevant variables within a classification framework [18]. For SVM-RFE, the candidate function and kernel type were “lrFuncs” and “svmLinear,” respectively. For elastic net, the regularization parameters *λ* and *α* were determined by fivefold cross-validation (*λ* = 0.09, *α* = 0.2). Here lambda (*λ*) is the penalty coefficient and alpha is for the elastic net mixing parameter *α*, with range [0–1]. Default parameters were used for other models. Accuracy was utilized as an evaluation indicator to determine the optimal features for each model. Then, the predictive performance of the models was validated in the validation cohort. Finally, the AUCs and Kappa values generated by bootstrapping for stratification were calculated using the “resampled” function in the Caret R package. Cohen's kappa is such a measure of inter-rater agreement for categorical scales when there are two raters [19]. The AUCs and kappa values were used to evaluate the accuracy of the stratification models.

### Statistical analysis

All statistical analyses were performed in *R* (version 4.1.0). Student’s *t*-test and Wilcoxon test were used to compare the difference between two sets of continuous variables. One-way analysis of variance and Kruskal–Wallis test was applied to conduct comparisons of three or more groups. Pearson’s *R* correlation was applied to calculate the correlation coefficient. The hazard ratio (HR) in the Cox regression model was calculated using the survival *R* package. The median follow-up period and its interquartile range were computed based on the reverse Kaplan–Meier method. *P*-values < 0.05 were considered statistically significant.

### Lasso regression

The Cox regression model with a Lasso penalty was used to establish the best model in the training cohort using the *R* package glmnet [20]. This process was repeated 1000 times to ensure the robustness of predictive PBIMs and the stability of the model. Then, the frequency of each model was calculated. We obtained the best regression model with the highest frequency, which was selected for the prognostic prediction model. Ultimately, four hub PBIMs with nonzero regression coefficients in the best gene model (with the highest frequency) were selected through Lasso regression model analysis with 1000 iterations, and the optimal lambda value was determined by tenfold cross-validation. Then, the BICS was constructed by the counts (absolute counts, and differential variations of the absolute counts) of the four hub PBIMs weighted by the multivariate Cox regression coefficient.

### ASR regression

The ASR regression is a model selection method that involves testing all possible compositions of PBIMs, and then choosing the best model based on the adjusted r-squared (R2_adj). Briefly, the “regsubsets” function (leaps package) was applied to identify different best models of different compositions of PBIMs. Here, the adjusted R2 demonstrates that the best model is the one with nine important variables.

## Results

### Patient characteristics of the study cohorts.

The detailed clinical characteristics of the ICI cohort are shown in Table 1. One hundred and twenty patients who received ICI with PD-1 inhibitor were enrolled in the ICI cohort, all of whom had whole blood samples taken for immunopheno-typing pre-ICI and before the first scan. The ICI cohort included 30 patients with non-small cell lung cancer (NSCLC), 22 patients with renal cell carcinoma (RCC), 16 patients with esophageal squamous cell carcinoma (ESCC), 13 patients with gastric adenocarcinoma (GAC), and 39 patients with other cancers (Fig. S1A). The median follow-up time was 14.6 months (range, 12.4–19.5 months). The anti-PD-1 drugs were sintilimab in 50 patients (65%), camrelizumab in 30 patients (23%), pembrolizumab in 20 patients (9%), nivolumab in 9 patients (2%), and others in 11 patients (1%) (Fig. S1B). Strikingly, we found that among the 65 patients who achieved SD at the first scan, approximately 40% (*n* = 26) did not ultimately reach DCB (Fig. 1B).

**Table 1:** Clinical characteristics of patients in the ICI cohort from 120 patients with metastatic cancer receiving ICI therapy

Features	Training cohort	Validation cohort	*P*-value
	(*N* = 91)	(*N* = 29)	
Sex			
Male	61.0 (67.0%)	20.0 (69.0%)	1
Female	30.0 (33.0%)	9.00 (31.0%)	
Age			
≤ 60	38.0 (41.8%)	15.0 (51.7%)	0.468
> 60	53.0 (58.2%)	14.0 (48.3%)	
Brain metastases			
No	83.0 (91.2%)	26.0 (89.7%)	1
Yes	8.00 (8.8%)	3.00 (10.3%)	
PD-L1			
< 1%	16.0 (17.6%)	2.00 (6.9%)	0.389
1%–49%	9.00 (9.9%)	0 (0%)	
50%–100%	7.00 (7.7%)	0 (0%)	
Missing	59.0 (64.8%)	27.0 (93.1%)	
Response			
PR	23.0 (25.3%)	9.00 (31.0%)	0.823
SD	50.0 (54.9%)	15.0 (51.7%)	
PD	18.0 (19.8%)	5.00 (17.2%)	
Clinical status			
DCB	52.0 (57.1%)	15.0 (51.7%)	0.407
NDB_LS	10.0 (11.0%)	6.00 (20.7%)	
NDB_SS	29.0 (31.9%)	8.00 (27.6%)	
Previous lines of treatment			
1	34.0 (37.4%)	13.0 (44.8%)	0.591
2	38.0 (41.8%)	8.00 (27.6%)	
3	14.0 (15.4%)	6.00 (20.7%)	
> 3	5.00 (5.5%)	2.00 (6.9%)	
PD-1 inhibitor type			
Camrelizumab	26.0 (28.6%)	4.00 (13.8%)	0.288
Nivolumab	6.00 (6.6%)	3.00 (10.3%)	
Others	10.0 (11.0%)	1.00 (3.4%)	
Pembrolizumab	14.0 (15.4%)	6.00 (20.7%)	
Sintilimab	35.0 (38.5%)	15.0 (51.7%)	

*DCB* durable clinical outcomes, *NDB* no durable benefit, *NDB_LS* long-term survival with no durable clinical benefit, *NDB_SS* short-term survival with no durable clinical benefit

The non-ICI cohort included 22 patients with NSCLC, 7 patients with small-cell lung cancer (SCLC), 4 patients with colorectal cancer (CRC), and 7 patients with other cancers. In the non-ICI cohort, 51% of the 20 patients reached DCB. The median follow-up time was 15.5 months (range, 10.6–23.9 months).

### PBIMs are associated with clinical outcomes

To analyze PBIMs and their correlation with clinical outcomes, patients in the ICI cohort were divided into a high and low group using the median value of PBIMs. The Kaplan–Meier plot revealed that four PBIMs, including differences in the variation of absolute T cell count (*p* = 0.0035, *p* = 0.0041), absolute lymphocyte count (*p* = 0.033, *p* = 0.046), absolute CD3 + CD4 + T cell count (*p* < 0.001, *p* = 0.0085), and absolute CD3 + CD8 + T cell count (*p* = 0.0065, *p* = 0.034), were significantly associated with PFS and OS (Fig. S2A and B). Patients with a highly differential variation of Treg cells (*p* = 0.0077), a low percentage of CD3 + CD8 + T cells before therapy, and a high absolute CD19 + B cell count before the first scan showed a shorter OS, suggesting that changes in peripheral biomarkers after initial treatment may also predict prognosis (Fig. S2B).

Additionally, we found that in NDB patients the absolute lymphocyte count, T cell count, CD19 + B cell count, CD3 + CD4 + T cell count, and CD3 + CD8 + T cell count showed a strong decrease in the peripheral blood, while DCB patients showed a significant increase in the absolute CD3 + CD4 + T cell count, lymphocyte count, T cell count, and CD3 + CD8 + T cell count (Fig. S3A).

### Construction of the BICS.

To uncover the practicability and accuracy of the BICS for patients with metastasized cancer treated with ICIs, the ICI cohort was divided randomly into the training (*n* = 91) and validation (*n* = 29) cohorts. The clinicopathological characteristics were comparable among the two cohorts (*p* > 0.05) (Table 1). After the data cleaning steps, in the training cohort, 43 PBIMs and the clinicopathological information were included in univariate Cox survival analysis. Finally, we found that five of them were associated with OS (*p* < 0.05) (Table S1). However, we observed high correlation and multicollinearity (variance inflation factor > 10) among some PBIMs (Fig. S4A), which would prejudice the results of traditional Cox regression analysis. To select optimal features, we applied two other well-established feature selection algorithms (ASR and Lasso regression). The ASR process with the “regsubsets” function was applied to identify the best nine-features model with the highest R2_adj (Fig. S5A). The Lasso process using the glmnet R package was repeated 1000 times to ensure the robustness of predictive PBIMs, and the stability of the model. After 1000 iterations, five emergent models and the associated PBIMs incorporated into the models were determined and are listed in Table S2. Then, the frequency of each model was calculated. As illustrated in Fig. S5B, we identified the best Lasso model, which had the highest frequencies compared to the other four models. We evaluated the performance of three models based on the indices of model performance, and the AUC through tenfold cross-validation. The different indices were normalized and the Lasso model indicates better model performance than the ASR and Cox regression models (Fig. 1C). The predictive value of the Lasso model exceeded the ASR and Cox regression models in both the time-dependent ROC curve at 12 and 24 months of OS (Fig. 1D, E). Finally, we obtained the best penalized regression model (Lasso) with the highest c-index compared to the two other penalized regressions (ridge regression and elastic net) (Fig. S5C). The final signature, named ‘BICS’ was computed for each patient formulated by the values and risk coefficient of each PBIM (Fig. S5D). The four hub PBIMs with nonzero regression coefficients in the best model are summarized in Table S3. The absolute values of the correlation coefficients of four variables in the Lasso model were < 0.5 (Fig. S5E), indicating that these four variables are no longer strongly correlated, and contain more information than noise.

### Prognostic and predictive values of BICS in response to ICIs

**Fig. 2:**
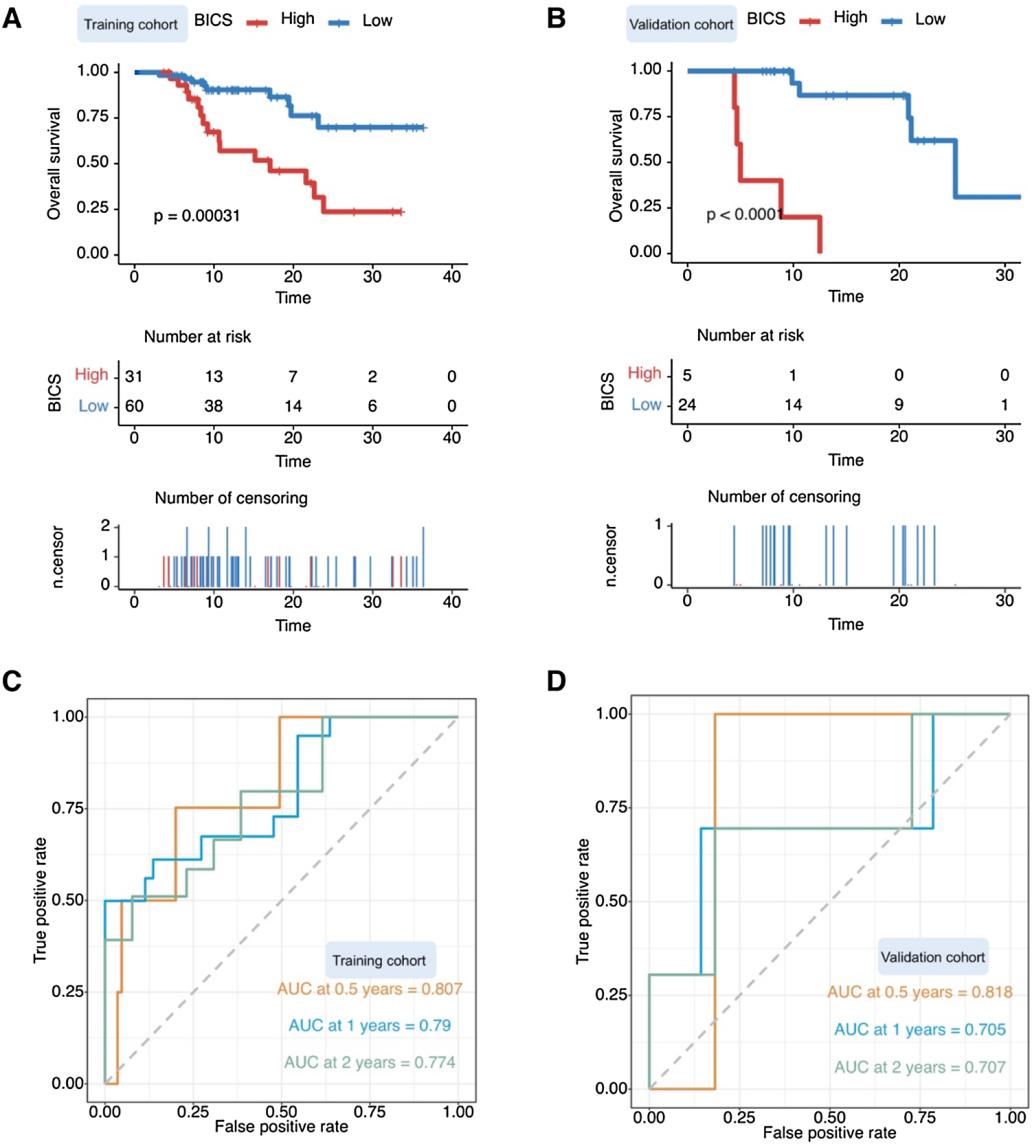
**A** Kaplan–Meier analysis showed that patients with a higher BICS exhibited a worse OS in the training cohort. **B** Kaplan–Meier analysis showed that patients with a higher BICS exhibited a worse OS in the validation cohort. **C** Time-dependent ROC curve at 0.5-, 1-, and 2-year OS for BICS in the training cohort (0.5-year AUC = 0.807, 1-year AUC = 0.79, and 2-year AUC = 0.774). **D** Time-dependent ROC curve at 0.5-, 1-, and 2-year OS for BICS in the validation cohort (0.5-year AUC = 0.818, 1-year AUC = 0.705, and 2-year AUC = 0.707). BICS: peripheral-blood-immune-cell-based signature; AUC: area under the curve

**Fig. 3:**
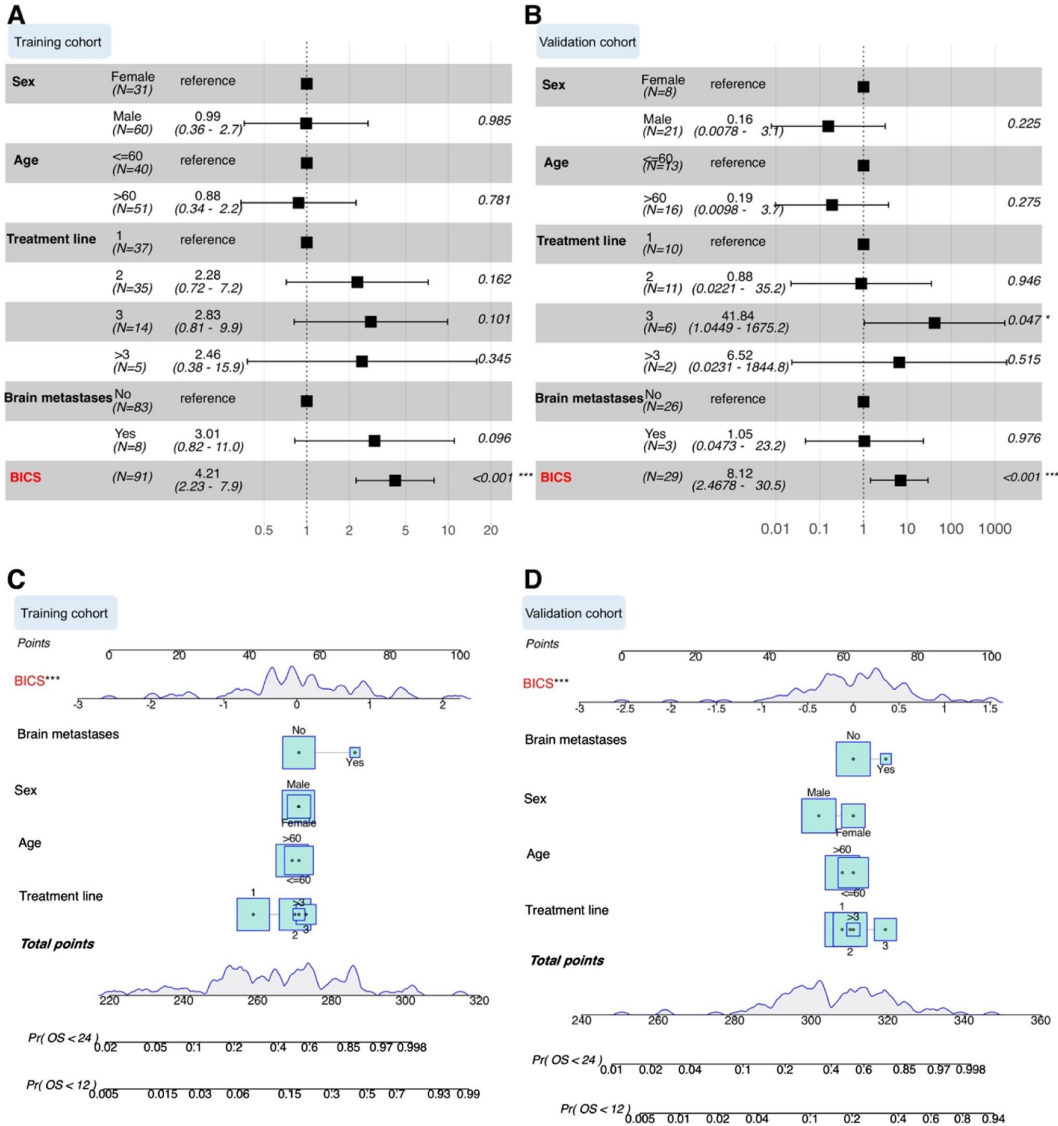
**A** Multivariate Cox analysis independently evaluating the predictive ability of BICS and other clinical characteristics for OS in the training cohort. The square data represent the estimated hazard ratios. The error bars indicate 95% CIs. **B** Multivariate Cox analysis independently evaluating the predictive ability of BICS and other clinical characteristics of OS in the validation cohort. The square data represent the estimated hazard ratios. The error bars indicate 95% CIs. **C** A nomogram was constructed with the training cohort for predicting the probability of 1-year and 2-year OS. **D** A nomogram was constructed with the validation cohort for predicting the probability of 1-year and 2-year OS. BICS: peripheral-blood-immune-cell-based signature

We classified the training cohort into two groups based on the BICS. In the training cohort, the Kaplan–Meier plot revealed that patients in the high-BICS group had a poorer PFS and OS than those in the low-BICS group (Fig. 2A and S6A). The BICS showed strong clinical significance for predicting progression based on the ROC curves in Fig. 2C (0.5-year: 0.807, 1-year: 0.79, and 2-year: 0.774). In line with the training cohort, Kaplan–Meier analysis demonstrated significant differential survival outcomes between the high- and low-BICS groups in the testing cohorts (Fig. 2B and S6B). Similarly, the BICS still exhibited efficacy in prognostic value assessment in the validation cohort (Fig. 2D). To investigate whether the prognostic value of the BICS is independent of other clinical factors, we performed multivariate Cox regression analysis. The results indicated that the BICS was remarkably associated with OS after adjustment for clinical characteristics, such as sex, age, treatment line, and brain metastases, thus confirming its robustness in independently predicting GC prognosis (hazard ratio [HR]: 4.21, 95% confidence interval [CI]: 2.23–7.9, *p* < 0.001) (Fig. 3A). A similar result was observed in the validation cohort (HR: 8.12, 95% CI: 2.46–30.5, *p* < 0.001) (Fig. 3B). We also established a nomogram based on the BICS, treatment line, age, sex, and brain metastases in the training cohort, which was also tested in the validation cohort (Fig. 3C, D). The BICS was found to contribute the most risk points compared to the other predictors (Fig. 3C, D).

**Fig. 4:**
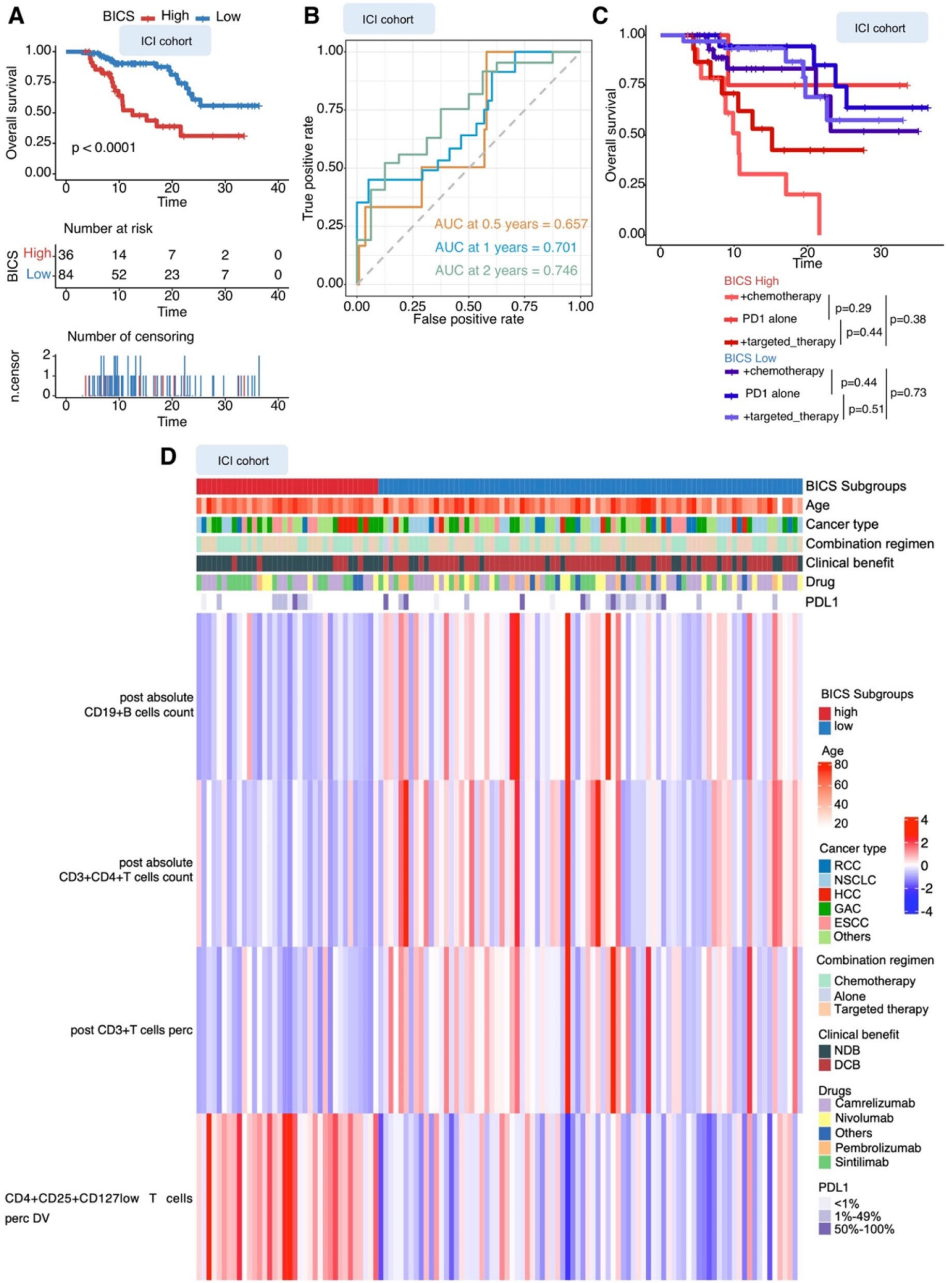
**A** Kaplan–Meier analysis showed that patients with a higher BICS exhibited a worse OS in the ICI cohort. **B** Time-dependent ROC curve at 0.5-, 1-, and 2-year OS for BICS in the ICI cohort (0.5-year AUC = 0.657, 1-year AUC = 0.701, and 2-year AUC = 0.746). **C** OS from the start of therapy stratified by BICS in patients in the ICI cohort treated with PD-1 single-agent blockade, PD-1 and chemotherapy combination therapy, or a combination of PD-1 and targeted therapy. **D** Clustered heatmap showing the four immune-related PBIMs that classify patients (represented in columns) into DCB or NDB groups. BICS: peripheral-blood-immune-cell-based signature; ICIs: immune checkpoint inhibitors; AUC: area under the curve; PD-1: programmed death 1; PD-L1: programmed death 1 ligand; DCB: durable clinical outcomes; NDB: no durable benefit; RCC: renal cell carcinoma; ESCC: esophageal squamous cell carcinoma; GAC: gastric adenocarcinoma; SCLC: small-cell lung cancer; NSCLC: non-small cell lung cancer; HCC: hepatocellular carcinoma

To evaluate whether the BICS predicts prognosis in non-ICIs patients, we additionally examined its association with outcomes in patients who received non-ICIs. Interestingly, there was no significant difference in PFS and OS between the high- and low-BICS subgroups in the non-ICI cohort (Fig. S6D, E). In the total ICI cohort, we confirmed that the BICS retained its ability to predict PFS and OS (Fig. 4A and S6C). Furthermore, ROC analysis showed that the BICS had excellent accuracy regarding 0.5-, 1-, and 2-year OS (AUC = 0.657, 0.701, and 0.746, respectively; Fig. 4B). In addition, patients with a high BICS had similarly reduced OS, regardless of treatment regiments, while patients with low BICS had equally prolonged OS (Fig. 4C). Strikingly, the BICS exhibited predictive value in patients who will achieve DCB (Fig. 4D). A combination of four variables was preferred to distinguish between the two clinical benefit groups: increased variations of Treg cells were associated with NDB patients, whereas an increased percentage of CD3 + T cells, absolute CD19 + B cells, and CD3 + CD4 + T cells before the first scan was related to DCB (Fig. 4D).

### BICS in patients with initial SD stratification

**Fig. 5:**
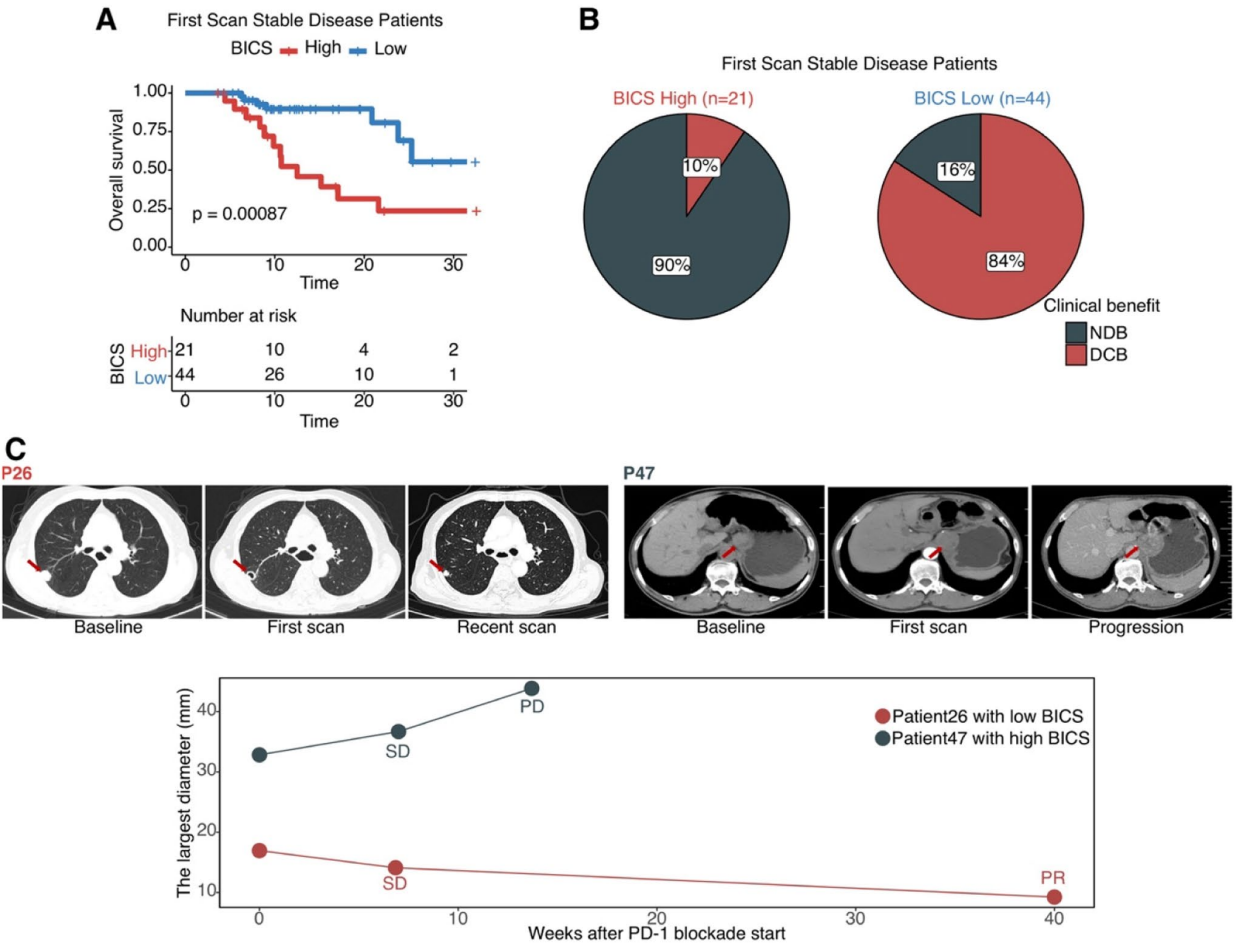
**A** Kaplan–Meier analysis showed that patients with RECIST SD at the first scan with a higher BICS exhibited a worse OS in the ICI cohort. **B** Pie charts demonstrate the proportions of patients with RECIST SD at the first scan. The analysis is split by those patients with a high BICS (*n* = 21) versus those with a low BICS (*n* = 44). **C** P26: Vignette for a patient with low BICS and SD at the first scan. P47: Vignette for a patient with high BICS and SD at the first scan. BICS: peripheral-blood-immune-cell-based signature; PD: progressive disease; PR: partial response; SD: stable disease; DCB: durable clinical outcomes; NDB: no durable benefit

In patients whose first scan after treatment initiation was classified as stable, high-BICS patients had a worse OS than low-BICS patients (Fig. 5A), and the low-BICS group accurately identified 84% of patients eventually achieving a DCB (Fig. 5B). The results of two representative patients emphasize that BICS can identify patients with SD at the first evaluation, who may obtain a DCB from ICI. P26 had a low BICS before the first scan, while P47 had a high BICS. As predicted by BICS, at 14 weeks after therapy initiation, P47 exhibited progression at the second scan, while P26 had a durable benefit from ICI and achieved radiographic PR at 40 weeks (Fig. 5C).

### Use of the SVM in predicting optimal clinical benefit

**Fig. 6:**
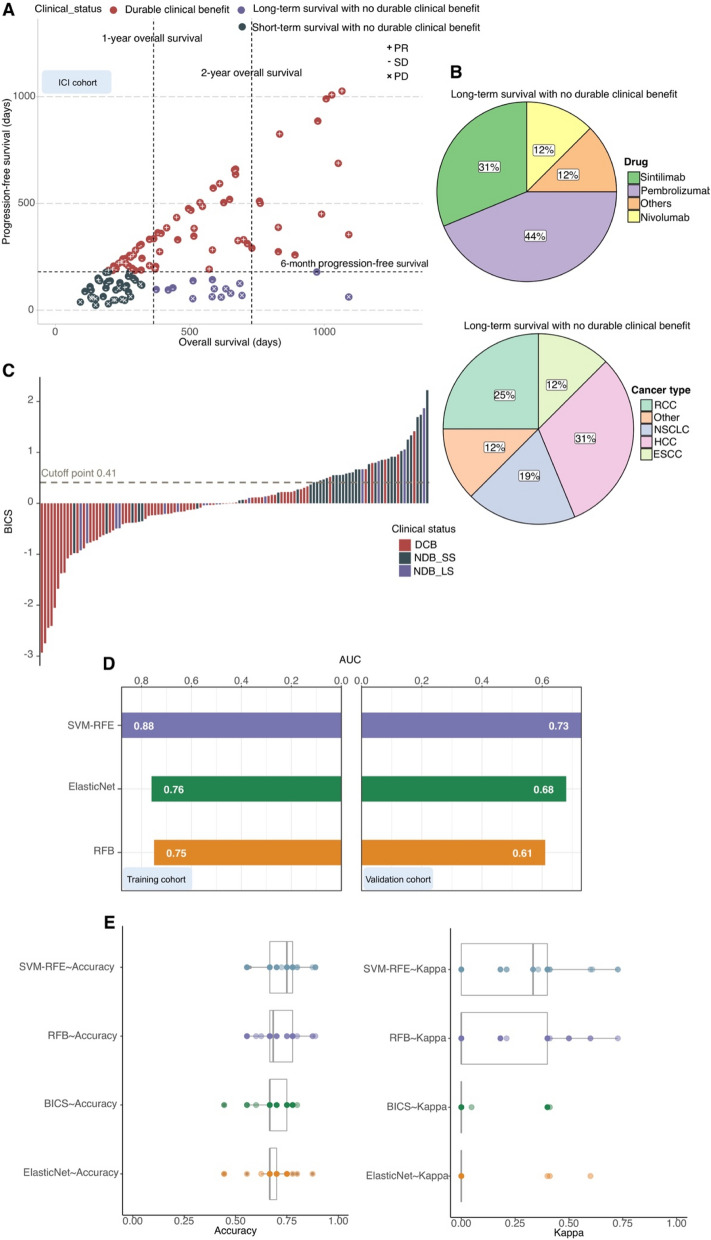
**A** Patients were stratified into response groups based on RECIST criteria (PR, SD, and PD), duration of OS, and duration of PFS. “Long-term survival with no durable clinical benefit (NDB_LS)” was defined as patients who achieved NDB with ICI therapy (OS > 1 year) (*n* = 37). An additional cohort of patients who achieved short-term survival (OS < 1 year) after ICI treatment with early tumor progression (PFS < 6 months) was considered separately (*n* = 16). **B** Pie charts demonstrating the proportions of NDB_LS patients. **C** Waterfall plot showing the correlation of BICS with clinical status (NDB_LS, NDB_SS, and DCB). BICS: peripheral-blood-immune-cell-based signature; DCB: durable clinical outcomes; NDB_LS: long-term survival with no durable clinical benefit; NDB_SS: short-term survival with no durable clinical benefit; NDB: no durable benefit; PD: progressive disease; PR: partial response; SD: stable disease; RCC: renal cell carcinoma; ESCC: esophageal squamous cell carcinoma; NSCLC: non-small cell lung cancer; HCC: hepatocellular carcinoma. **D** Bar plot showing the AUC of each model for classifying NDB versus NDB_LS& DCB in both the training and validation cohorts. **E** The accuracy and kappa generated by bootstrapping for classifying NDB versus NDB_LS& DCB using the SVM-RFE, RFB, elastic net, and BICS models. BICS: peripheral-blood-immune-cell-based signature; SVM-RFE: support vector machine-recursive and feature elimination; RFB: random forest and Boruta

To stratify our ICI cohort, “long-term survival with no durable clinical benefit (NDB_LS)” was defined using a composite end point of NDB with OS > 1 year; these patients showed early progression from ICI (PFS < 6 months), but their OS exceeded 1 year (*n* = 16) (Fig. 6A). “Short-term survival with no durable clinical benefit (NDB_SS)” was defined as NDB with OS < 1 year (*n* = 37) (Fig. 6A). NDB_LS patients were independent of anti-PD-1 regiments and cancer types (Fig. 6B). These NDB_LS and DCB patients were collectively referred to as “optimal clinical benefit” and they achieved a long PFS or an OS benefit from ICI therapy.

Unfortunately, BICS showed low values in optimal clinical benefit patient stratification (Fig. 6C). To address the patients with optimal clinical benefit stratification, we developed a series of machine learning algorithms (Materials and Methods). First, these machine learning algorithms were used to perform a dimension reduction to reduce noise or redundant features. Based on the RFB, it was concluded that four important features affect the final stratification (Fig. S7A). The RFE recommends six features for the model with the highest level of accuracy (Fig. S7B–C). The elastic net model, using 20 of the 43 features, provides an impressive segregation between optimal clinical benefit and non-optimal clinical benefit (Fig. S7D). We further evaluated the classification performance of the SVM-RFE, RFB, and elastic net models and found that the SVM-RFE model outperformed other models in both the training and validation cohorts (Fig. 6D). In the ICI cohort, the bootstrapping-generated accuracy and kappa value of optimal clinical benefit classification were compared across the SVM-RFE, RFB, elastic net, and BICS models. Consistent with the AUC comparison performance, the SVM-RFE model had significantly higher accuracy and kappa value compared to other models (Fig. 6E).

## Discussion

Compared to traditional therapies, ICI treatments have demonstrated durable responses in various malignancies. However, not all patients experience durable responses, or prolonged survival in response to ICIs [21]. Recent analysis of TMB and tumor-infiltrating immune cells gene expression has provided value in recognizing patients most likely to respond to pembrolizumab, indicating the prospective value of these biomarkers in the selection of patients for immunotherapy [6, 22]. Although tumor biopsies are broadly used for assessing tumor PD-L1 expression and TMB, obtaining tissue can be challenging due to the heterogeneity of biopsy sites, risk of adverse events, and limited accessibility. In addition, some trials have shown that PD-L1 expression has an unsatisfactory predictive power, and, as such, the TMB still undergoes clinical evaluation [6, 23]. Given that the availability of predictive biomarkers is limited, there is a pressing need to identify a prognostic biomarker for immunotherapy. As one of the readily accessible biomarkers, PBIMs enables early assessment of treatment response, which will facilitate early changes in management. Our study compared and integrated the differential variations of each PBIM before and after therapy, and subsequently included these differential variations as variables in the regression models. Despite data cleaning, complex patterns of correlation between variables still existed. The high correlation present in the independent variables leads to an expansion of the standard errors of the regression coefficients, which makes the current regression model unstable [24]. The regularization of the coefficients can be applied to overcome the overfitting problem caused by multicollinearity [25]. Through the comparison of a series of machine learning algorithms, Lasso, as a type of regularized regression, can better limit the impact from multicollinearity. We utilized the Lasso Cox algorithm to identify immune-related PBIMs affecting OS and constructed a BICS based on PBIMs. The BICS proved to be a valid prognostic immune-related biomarker for patients with metastatic cancer, with better survival observed in BICS-low patients and worse survival in BICS-high patients in our ICI cohort.

The BICS comprised four PBIMs, including different variations in the percentages of Treg cells, CD3 + T cells, and the absolute CD19 + B cell and CD3 + CD4 + T cells counts. Indeed, alterations in Treg number and function have been previously described in patients receiving immunotherapy [26, 27]. Several studies have linked the accumulation of Tregs to poor prognosis due to suppression of the antitumor immune response [14, 28, 29]. A decrease in early CD19 + B cells was also observed in NDB patients in our study, highlighting that B cell monitoring might more accurately identify patients who will achieve NDB. In a study of patients with non-small-cell lung cancer, flow cytometry and RNA analysis revealed that the percentage of circulating CD4 + and CD8 + T cells correlated with inflamed tumors, indicating that all of these markers play an important role in the anti-tumor responses [30]. In the calculation formula of the BICS, the coefficients of Treg cells were negative, while the coefficients of CD3 + T cells, CD19 + B cells, and CD3 + CD4 + T cells were positive. Therefore, there was a negative relationship between BICS and CD3 + T cells, CD19 + B cells, and CD3 + CD4 + T cells, and a positive relationship between BICS and Treg cells. These data are consistent with the utility of these PBIMs in influencing the prognosis of metastatic cancer and the response to therapy in the ICI cohort. In conclusion, the BICS is a novel peripheral blood biomarker correlated with immunosuppression and tumor progression.

The BICS showed an early, robust, and non-invasive classification of DCB and NDB in a training and validation approach. In addition, the BICS performed well in different subgroups of patients, such as those receiving various PD-1 treatment types, anti-PD-1 therapies with chemotherapy, or targeted-therapy. More importantly, patients with SD at the first efficacy assessment were better classified using BICS. The immune response elicited by immunotherapy prevents conventional imaging from accurately assessing its efficacy [31]. Indeed, 40% of patients (26/65) who achieved SD at the first scan eventually underwent NDB, demonstrating that objective responses evaluated by radiographic images do not accurately capture patients who are gaining benefit. The BICS consists of a range of immune cells and serves as an effective tool to identify patients with SD at the first scan who will benefit from ICI treatment in the long term.

Several clinical trials have demonstrated that immunotherapy significantly improves long-term OS, but does not influence PFS [32, 33]. Consequently, we aimed to combine the ICIs efficacy assessment and prognostic prediction. The designation “NDB_LS” is derived from the above clinical trials in which some patients progressed early from ICI therapy (PFS < 6 months) but had an OS pattern lasting more than 1 year. The final aim of ICI therapy in patients with metastatic cancer is to yield long-term durable responses. There is an urgent need to recognize patients who may achieve optimal clinical benefit from ICI therapy (NDB_LS and DCB) and to identify the non-durable responders (NDB_SS) for alternative anti-tumor options. We applied a series of machine learning models with the integration of PBIMs to identify which patients will achieve optimal clinical benefit from ICIs. Zhou and colleagues previously reported a prognostic model that was trained with immune cell profiles from the peripheral blood of multitype advanced cancer patients [34]. In the same notion, although differences in cancer types, our SVM-RFE model has shown unexpected performance in the ICI cohort, indicating that PBIMs influence immune function systemically and independently of cancer type. Moreover, a recent report suggests that the prediction can be improved when integrating circulating immune cell profiling and ctDNA [35]. Therefore, we expect that the performance of our model can be further improved by incorporating other peripheral immune-related analytes, including soluble plasma proteins, ctDNA, circulating tumor cells, and cytokines.

Our study has some limitations. First, this was a retrospective analysis, which included patients with different types of cancer who were exposed to heterogeneous PD-1 regimens. Nevertheless, we observed that the BICS exhibited similar performance regardless of the PD-1 regimen and tumor type. Second, we validated the BICS and SVM-RFE models in a relatively small number of patients, resulting in relatively broad CIs. Therefore, further validation in prospective and multicenter clinical trials will be necessary. Third, using flow cytometry alone may not capture the dimensionality of the factors responsible for the ICI response. Therefore, the application of high-dimensional single-cell technologies to the analysis of cancer immunotherapy will be necessary. Finally, the efficacy of the BICS and classical immunotherapy-related markers was not assessed due to the lack of MSI, PD-L1 protein expression, and TMB-related metrics. We will attempt to test this comparison in future studies.

In conclusion, our study provides evidence that PBIMs represent both prognostic and predictive factors for outcomes. Moreover, our newly developed BICS based on early response assessment can stratify metastatic cancer patients into subgroups with different prognoses and diverse responses to ICI therapy. In addition, we established the SVM-RFE classifier model with moderate performance in predicting optimal clinical benefit (NDB_LS and DCB), which may improve the personalization of immunotherapy for patients with metastatic cancers.

## Supplementary Information

Below is the link to the electronic supplementary material.

Supplementary file1 (XLSX 64 KB)

## Data Availability

The datasets used and/or analyzed during the current study are available from the corresponding author on reasonable request.

## Abbreviations

AIC: Akaike’s information criterion
ASR: All-subsets regression
AUC: Area under the curve
BC: Breast cancer
BIC: Bayesian information criterion
BICS: Peripheral-blood-immune-cell-based signature
CI: Confidence interval
CRC: Colorectal cancer
CSCC: Cutaneous squamous cell carcinoma
CUP: Cancers of unknown primary
DCB: Durable clinical outcomes
DV: Differential variations
ESCC: Esophageal squamous cell carcinoma
GAC: Gastric adenocarcinoma
HCC: Hepatocellular carcinoma
HR: Hazard ratio
ICIs: Immune checkpoint inhibitors
LASSO: Least absolute shrinkage and selection operator
MSI: Microsatellite instability
NDB: No durable benefit
NDB_LS: Long-term survival with no durable clinical benefit
NDB_SS: Short-term survival with no durable clinical benefit
NSCLC: Non-small cell lung cancer
OC: Ovarian cancer
OS: Overall survival
PBIMs: Peripheral blood immune cell markers
PBMCs: Peripheral blood mononuclear cells
PD-1: Programmed death 1
PD-L1: Programmed death 1 ligand
PD: Progressive disease
PFS: Progression-free survival
PR: Partial response
R2: R-squared value
R2_adj: Adjusted r-squared
RCC: Renal cell carcinoma
RECIST: Response evaluation criteria in solid tumors
RFB: Random forest and Boruta
RMSE: Root-mean-squared error
ROC: Receiver operating characteristic
SCLC: Small-cell lung cancer
SVM-RFE: Support vector machine-recursive and feature elimination
TMB: Tumor mutation burden

## References

[1] Ramos-Casals M, Brahmer JR, Callahan MK, Flores-Chávez A, Keegan N, Khamashta MA (2020). Immune-related adverse events of checkpoint inhibitors. Nat Rev Dis Prim.

[2] Gavrielatou N, Doumas S, Economopoulou P, Foukas PG, Psyrri A (2020). Biomarkers for immunotherapy response in head and neck cancer. Cancer Treat Rev.

[3] Gibney GT, Weiner LM, Atkins MB (2016). Predictive biomarkers for checkpoint inhibitor-based immunotherapy. Lancet Oncol.

[4] Hecht M, Büttner-Herold M, Erlenbach-Wünsch K, Haderlein M, Croner R, Grützmann R (2016). PD-L1 is upregulated by radiochemotherapy in rectal adenocarcinoma patients and associated with a favourable prognosis. Eur J Cancer (Oxford, England: 1990).

[5] Bilgin B, Sendur MA, Bülent Akıncı M, Şener Dede D, Yalçın B (2017). Targeting the PD-1 pathway: a new hope for gastrointestinal cancers. Curr Med Res Opin.

[6] Cristescu R, Mogg R, Ayers M, Albright A, Murphy E, Yearley J (2018). Pan-tumor genomic biomarkers for PD-1 checkpoint blockade-based immunotherapy. Science.

[7] Grasso CS, Tsoi J, Onyshchenko M, Abril-Rodriguez G, Ross-Macdonald P, Wind-Rotolo M (2020). Conserved interferon-γ signaling drives clinical response to immune checkpoint blockade therapy in melanoma. Cancer Cell.

[8] Raskov H, Orhan A, Christensen JP, Gögenur I (2021). Cytotoxic CD8(+) T cells in cancer and cancer immunotherapy. Br J Cancer.

[9] Ganesh K, Stadler ZK, Cercek A, Mendelsohn RB, Shia J, Segal NH (2019). Immunotherapy in colorectal cancer: rationale, challenges and potential. Nat Rev Gastroenterol Hepatol.

[10] Wei C, Chen M, Deng W, Bie L, Ma Y, Zhang C (2022). Characterization of gastric cancer stem-like molecular features, immune and pharmacogenomic landscapes. Brief Bioinform.

[11] Nixon AB, Schalper KA, Jacobs I, Potluri S, Wang IM, Fleener C (2019). Peripheral immune-based biomarkers in cancer immunotherapy: can we realize their predictive potential?. J Immunother Cancer.

[12] Kim KH, Cho J, Ku BM, Koh J, Sun JM, Lee SH (2019). The first-week proliferative response of peripheral blood PD-1(+)CD8(+) T cells predicts the response to Anti-PD-1 therapy in solid tumors. Clin Cancer Res Off J Am Assoc Cancer Res.

[13] Li S, Zhang C, Pang G, Wang P (2020). Emerging blood-based biomarkers for predicting response to checkpoint immunotherapy in non-small-cell lung cancer. Front Immunol.

[14] Deng L, Zhang H, Luan Y, Zhang J, Xing Q, Dong S (2010). Accumulation of foxp3+ T regulatory cells in draining lymph nodes correlates with disease progression and immune suppression in colorectal cancer patients. Clin Cancer Res.

[15] Eisenhauer EA, Therasse P, Bogaerts J, Schwartz LH, Sargent D, Ford R (2009). New response evaluation criteria in solid tumours: revised RECIST guideline (version 1.1). Eur J Cancer.

[16] Lüdecke D, Ben-Shachar MS, Patil I, Waggoner P, Makowski D (2021). Performance: an R package for assessment comparison and testing of statistical models. J Open Sour Softw.

[17] Sanz H, Valim C, Vegas E, Oller JM, Reverter F (2018). SVM-RFE: selection and visualization of the most relevant features through non-linear kernels. BMC Bioinform.

[18] Kursa MB, Rudnicki WR (2010). Feature selection with the Boruta package. J Stat Softw.

[19] McHugh ML (2012). Interrater reliability: the kappa statistic. Biochem Med.

[20] Friedman J, Hastie T, Tibshirani R (2010). Regularization paths for generalized linear models via coordinate descent. J Stat Softw.

[21] Schoenfeld AJ, Hellmann MD (2020). Acquired resistance to immune checkpoint inhibitors. Cancer Cell.

[22] Chong W, Shang L, Liu J, Fang Z, Du F, Wu H (2021). m(6)A regulator-based methylation modification patterns characterized by distinct tumor microenvironment immune profiles in colon cancer. Theranostics.

[23] Camidge DR, Doebele RC, Kerr KM (2019). Comparing and contrasting predictive biomarkers for immunotherapy and targeted therapy of NSCLC. Nat Rev Clin Oncol.

[24] Daoud JI (2017). Multicollinearity and regression analysis. J Phys Conf Ser.

[25] Ranstam J, Cook JA (2018). LASSO regression. British J Surg.

[26] Kavanagh B, O'Brien S, Lee D, Hou Y, Weinberg V, Rini B, Allison JP, Small EJ, Fong L (2008). CTLA4 blockade expands FoxP3+ regulatory and activated effector CD4+ T cells in a dose-dependent fashion. Blood.

[27] Alice Long S, Buckner JH (2011). CD4+FOXP3+T regulatory cells in human autoimmunity: more than a numbers game. J Immunol.

[28] Wolf D, Wolf AM, Rumpold H, Fiegl H, Zeimet AG, Muller-Holzner E (2005). The expression of the regulatory T cell–specific forkhead box transcription factor FoxP3 is associated with poor prognosis in ovarian cancer. Clin Cancer Res.

[29] Farinha P, Al-Tourah A, Gill K, Klasa R, Connors JM, Gascoyne RD (2010). The architectural pattern of FOXP3-positive T cells in follicular lymphoma is an independent predictor of survival and histologic transformation. Blood.

[30] Manjarrez-Orduño N, Menard LC, Kansal S, Fischer P, Kakrecha B, Jiang C (2018). Circulating T cell subpopulations correlate with immune responses at the tumor site and clinical response to PD1 inhibition in non-small cell lung cancer. Front Immunol.

[31] Anagnostou V, Forde PM, White JR, Niknafs N, Hruban C, Naidoo J (2019). Dynamics of tumor and immune responses during immune checkpoint blockade in non-small cell lung cancer. Can Res.

[32] Schadendorf D, Hodi FS, Robert C, Weber JS, Margolin K, Hamid O (2015). Pooled analysis of long-term survival data from phase II and Phase III trials of Ipilimumab in unresectable or metastatic melanoma. J Clin Oncol Off J Am Soc Clin Oncol.

[33] Schadendorf D, Fisher DE, Garbe C, Gershenwald JE, Grob JJ, Halpern A (2015). Melanoma. Nat Rev Dis Prim.

[34] Zhou JG, Donaubauer AJ, Frey B, Becker I, Rutzner S, Eckstein M (2021). Prospective development and validation of a liquid immune profile-based signature (LIPS) to predict response of patients with recurrent/metastatic cancer to immune checkpoint inhibitors. J Immunother Cancer.

[35] Nabet BY, Esfahani MS, Moding EJ, Hamilton EG, Chabon JJ, Rizvi H (2020). Noninvasive early identification of therapeutic benefit from immune checkpoint inhibition. Cell.

